# Musical improvisation enhances interpersonal coordination in subsequent conversation: Motor and speech evidence

**DOI:** 10.1371/journal.pone.0250166

**Published:** 2021-04-15

**Authors:** Juan Pablo Robledo, Sarah Hawkins, Carlos Cornejo, Ian Cross, Daniel Party, Esteban Hurtado

**Affiliations:** 1 Centre for Music and Science, University of Cambridge, Cambridge, Cambridgeshire, United Kingdom; 2 Laboratoire Interpsy EA4432, Université de Lorraine, Nancy, Lorraine, France; 3 Laboratorio de Lenguaje, Interacción y Fenomenología (LIF), Escuela de Psicología, Pontificia Universidad Católica de Chile, Santiago, Región Metropolitana, Chile; 4 Instituto de Música, Pontificia Universidad Católica de Chile, Santiago, Chile; Northeastern University, UNITED STATES

## Abstract

This study explored the effects of musical improvisation between dyads of same-sex strangers on subsequent behavioural alignment. Participants–all non-musicians–conversed before and after either improvising music together (Musical Improvisation—MI—group) or doing a motoric non-rhythmic cooperative task (building a tower together using wooden blocks; the Hands-Busy—HB—group). Conversations were free, but initially guided by an adaptation of the Fast Friends Questionnaire for inducing talk among students who are strangers and meeting for the first time. Throughout, participants’ motion was recorded with an optical motion-capture system (Mocap) and analysed in terms of speed cross-correlations. Their conversations were also recorded on separate channels using headset microphones and were analysed in terms of the periodicity displayed by rhythmic peaks in the turn transitions across question and answer pairs (Q+A pairs). Compared with their first conversations, the MI group in the second conversations showed: (a) a very rapid, partially simultaneous anatomical coordination between 0 and 0.4 s; (b) delayed mirror motoric coordination between 0.8 and 1.5 s; and (c) a higher proportion of Periodic Q+A pairs. In contrast, the HB group’s motoric coordination changed slightly in timing but not in degree of coordination between the first and second conversations, and there was no significant change in the proportion of periodic Q+A pairs they produced. These results show a convergent effect of prior musical interaction on joint body movement and use of shared periodicity across speech turn-transitions in conversations, suggesting that interaction in music and speech may be mediated by common processes.

## Introduction

Comparative approaches to music and language have revealed an increasing number of apparent commonalities [1], one of them being temporal organization. Indeed, the perception of both speech and music share the temporal organization of acoustic signals as foundational [2–4]. Yet comparative approaches often rely more or less directly on the idea of music and language as discrete modules or domains [5, 6]. This dichotomy is increasingly undermined by biological and cognitive evidence for overlap [7] as well as by the lack of any clear demarcation between music and language in many societies [8–10]. An alternative way to understand the commonalities between speech and music is to conceive of them not as distinct human capacities or domains that draw on common central resources, but as interactive media that are culture-specific manifestations of a common human communicative ‘toolkit’ of underlying cognitive and behavioural resources [11].

In this view, language [12] and music [13] are conceived of as fundamentally social, with the human capacity for flexible interaction with others as key to understanding much human cognition and behaviour. Complex sociality appears to be a human predisposition [14, 15], and it has been suggested that “[…] social interaction could be the default mode via which humans communicate with their environment” [16], p.181. This suggestion is increasingly supported by cognitive and neuroscientific evidence indicating, for instance, that aspects of self-other differentiation and control are primary mechanisms governing social cognition and behaviour—e.g., [17]. An interactionist perspective on music and language—or more accurately, speech (see, e.g., [18, 19])—provides a way of resolving apparent paradoxes that arise when speech and music are treated as individual capacities and as clearly distinct domains.

Consequently, we can expect that features that characterize interaction in speech could appear in music, and vice versa, when interactions switch between the two, at least when their sociocultural context and function, or register, remain similar [20]. Besides, music and speech are based on largely the same universal suite of human communicative capacities, crossmodal effects should apply not only to trained musicians but also populations without formal musical training. Moreover, if social interaction is a basic component of the human communicative ‘toolkit’, effects across domains should be evident not only in perception but also in production competencies involving social alignment. These considerations form the background to the experiment reported in this paper.

In order to present the specific rationale for the present study, the following sections define and review the notion of alignment in communicative interaction, as well as the two dimensions of interactional alignment that this study explores: motor alignment, which involves coordination of body movement between individuals, and speech alignment, which involves convergence between interlocutors of relevant acoustic parameters of speech.

### Communicative interaction and alignment

Interpersonal alignment has been understood as a natural tendency in humans to reduce differences in multimodal communication patterns when interacting with a partner [21–28]. Referred to by different terms, alignment patterns have been reported at different levels such as psychophysiological, neurophysiological, linguistic and motoric. For instance, psychophysiological research in contexts of natural interaction and play show that in certain circumstances people coordinate their breathing [29], heartbeats [30] and galvanic skin response [31]. These phenomena have been termed “dyadic synchronization” [30]. Similarly, neurophysiological studies find evidence of coupled neural activity between pairs of individuals solving imitative or coordinative tasks together, variously calling the process “interbrain coupling” [32], “brain coherence” [33] and “interbrain synchronization” [34]. Linguistic studies also show “interactive alignment” or “speech convergence” at multiple scales of linguistic structure. For example, during spontaneous conversation, interactants align their regional accent, lexicon and physical properties of pauses and speech e.g., rate, duration and frequency of occurrence when the conversational turn is aligned [35, 36] but not when it is not [37]. People spontaneously display temporally-synchronized patterns of body movement during social exchanges, variously termed “behavior matching” [38], “motor alignment” [39], “interpersonal coordination” [40] or, in cases where the joint behaviour is organised around a temporal periodicity, “entrainment” [41].

Despite the contribution of these studies, the tendency to work on parallel tracks in behavioural, neuroscientific and linguistic research, along with the use of diverse and often alternative terminology, has made it difficult to move towards a more comprehensive understanding of alignment phenomena in face-to-face human interactions. Nonetheless, studies of interpersonal alignment at more than one analytic level consistently show close correspondences between speech, facial gesture and other body movement during conversation [38, 42, 43] especially in affiliative rather than argumentative contexts [44]. Importantly, alignment of visual with acoustic cues significantly benefits speech intelligibility, especially in adverse listening conditions [45]. D’Ausilio and collaborators’ [46] review evidence for similar alignment patterns in a variety of scripted ensemble musical performances.

Musical interaction, whether scripted or improvisatory, typically produces sequences of events with inter-event durations that are experienced as related by simple ratios; for example, the duration of an event may be perceived as half, or twice, or one-third, or three times (and so on) the duration of the event that succeeds or precedes it [47], allowing a train of events to be apprehended as temporally patterned or rhythmic. Perception of a rhythmic sequence may give rise to the experience of a steady, periodic pulse, even if a pulse is not phenomenally present in the sequence of events [48] and people interacting in music will tend to organise their contributions around such an inferred, shared pulse [41, 49]. Those contributions need not be synchronous [50], but they will be temporally entrained to the shared pulse (interestingly, even music that is claimed to exhibit “free rhythm” has been shown to be organised around a steady pulse; see [51, 52].

In a departure from the trend to explore alignment in just one interactive domain, Hawkins, Cross and Ogden [49] examined evidence for alignment across speech and music. They found greater alignment between spoken and musical pulse when interactants fluently improvised music together compared with when improvisation was less fluent. These and other studies have opened a research field that tests the idea that common processes underpin interpersonal alignment in different domains [49, 53–57].

### Motor alignment

Motor Alignment (MA), also called ‘interpersonal coordination’ [58, 59], is understood as the degree of temporal synchronization between people’s body movements during a social interaction [27, 60]. Early MA studies used video analysis to describe temporally-synchronized motion patterns among participants during natural interaction. For example, micro-patterns of synchronized communicative behaviours were identified among conversing adults [25], and between adult speech and newborns’ movements [23]. Behavioural coding of video-recorded interactions showed associations between synchronized global behaviour and interactants’ positive affective states [61]. Since video analysis is labour-intensive and often hard to quantify, it has limited value in examining the dynamics underlying body-coordinating phenomena [62].

Recent motion tracking systems allow faster and more reliable spatial and temporal data capture [58]. Devices such as accelerometers, potentiometers, electrogoniometers, optical and magnetic capture systems have been used to record movements of people trained to perform tasks requiring synchronization with a referent (e.g., a metronome, a video, confederate or another participant). Movements of markers attached to body parts or moveable objects (e.g., pendulums) may also be tracked. Studies using such devices have not only deepened understanding of the physical characteristics of MA (temporal scales, velocities and angles), but have also allowed the parameters that underpin MA and resultant outcomes to be described in detail [63–65]. For instance, amongst biomechanical variables that influence MA, synchronization increases when: in-phase movement is predominant [40]; the preferred periodicity of movement is similar between interactants [66]; dyads move pendulums of similar length [66]; and interactants have visual, auditory or haptic access to their partner [59, 67]. Psychosocial studies show that MA is enhanced when people interact with a pro-social [68], physically attractive [65] or socially competent partner [69]. Conversely, MA decreases when the interactive partner has been diagnosed with mental or developmental disorders [70]. Studies of social-environmental factors show that simultaneous coordination increases in competitive and recreational interactive contexts and, to a lesser extent, in collaborative contexts. Conversely, MA tends to be reduced in emotionally negative or conflicting contexts. Finally, reports on psychological outcomes of MA suggest that body synchronization increases compassion and prosocial behaviour [71], self-esteem [63], trust [72], empathy and affiliation [61, 73]. However, the highly structured and unnatural tasks that are often introduced to control experimental variables may jeopardise the ecological validity, and hence the generalisability, of these findings [74].

Recent MA studies of interaction have introduced more natural conversational and musical contexts with some success. They have shown, for example, more coordination between strangers conversing affiliatively than argumentatively [44], and that while synchronization arises during affiliative conversations between friends and between strangers, it is significantly greater between friends [75]. Ellamil et al. [76], investigating influences of various musical features on synchronized movements amongst people dancing together in groups of six, found that group synchrony was associated with song popularity and with musical beats similar to a walking pace. Likewise, using silent disco technology, Woolhouse et al. [77] showed enhanced memory for physical attributes of participants who had danced to the same music compared to memory for other participants who had also danced together in pairs, but while the music they heard had very different tempi.

Despite their importance, naturalistic studies are still limited by a lack of accurate measurements of people’s movements while participating in spontaneous conversations and musical interactions. Although studies in conversational and musical contexts have provided promising perspectives, a more detailed understanding of MA requires more accurate measurement of movement in naturalistic interaction involving speech and music than has been achieved to date [58].

### Temporal alignment in speech

“Alignment” is often used in the speech literature to refer to phenomena such as evidence of enhanced understanding of or attitude towards topic without reference to the temporal domain [78]. In fact, many linguistic and phonetic parameters, including lexis, syntax, duration, loudness, and pitch converge between interactants during affiliative talk [79]. Here, we restrict our focus on speech alignment (SA) mainly to temporal parameters. This focus is consistent with the hypothesis that greater rhythmic coordination between interactants indicates stronger affiliative behaviour as argued for musical interaction by Cross [80] and inferred, for example, by Trainor & Cirelli [81] and Cirelli et al. [82], who showed increased prosocial behaviour following synchronous rhythmic movement. It is also consistent with the literature most relevant to the present research.

We have introduced the idea that, as dynamic joint activities in which interactants must coordinate their individual actions in order to succeed [83], conversation and ensemble music-making both exhibit entrainment to a shared regular pulse as a central emergent property [84]. By analysing “pikes”—local f0 maxima or minima on accented syllables [85]—and musical pulse produced by dyads of native English speakers who talked while they improvised music, Hawkins, Cross and Ogden [49] concluded that interactants seem to entrain to one another over short periods regardless of domain. Such entrainment seems to produce SA (speech alignment): gestures or vocalisations interpretable as supporting the flow of current conversation [78]. Similarly, building on and refining Couper-Kuhlen’s [86] work in Conversation Analysis (CA) on temporal alignment in preferred and dispreferred answers to questions, Ogden and Hawkins [54] compared instances of shared periodicity vs. aperiodicity in Q+A pairs spoken by the same native-English-speaking dyads during conversations that did not include music-making. (Though note that, following Couper-Kuhlen [86]), they used the terms “rhythmic” and “arhythmic” rather than “periodic” and “aperiodic”.) Ogden and Hawkins concluded that the ends of well-formed questions typically become roughly periodic (“rhythmic” in their paper), in that intervals between successive pikes have similar durations; and that when the answers that follow such periodic questions are also well-formed, in the CA sense of being preferred, they maintain that same periodicity when they enter the turn space. In short, time intervals between successive pikes become roughly periodic across turn transitions in well-formed Q+A pairs. This periodicity is a locally-available pragmatic resource strongly related to social preference, which facilitates turn-taking and the interpretation of the answerer’s intention by increasing SA. These findings suggest a close connection between prosodic and musical behaviour. Though this suggestion is not new (speech prosody has long been seen as musical), Robledo et al. [87] offer insights into how these close connections can work. Temporal and (absolute and relative) pitch measures operate across rather than just within turns. They work together: pragmatically-aligned interactions can involve short stretches of strongly-entrained, periodic rhythm and quite precise enculturated pitch intervals; disaligned (dispreferred) interactions do not.

### Rationale for the present study

The evidence reviewed above regarding body movement and speech alignment during music-making and talking suggests, first, that successful musical and linguistic interaction demand entrainment and reciprocal interpersonal alignment, and second, that reciprocal alignment is indicative of, and gives rise to, affiliative attitudes. These points are relatively uncontroversial. However, several questions must be answered before the processes involved can be well understood. Foremost amongst these are (1) whether the periodicity across conversational turns observed for English applies also to conversants speaking a language whose prosody differs markedly from that of English; (2) whether periodicity is affected when the conversants do not know one another; and (3) to what extent the particular ‘joint-action’ task—in particular its inherent rhythmicity—affects results.

The study reported here set out to answer these questions by extending the work of Hawkins, Cross and Ogden [49] and Ogden and Hawkins [54] in four ways. First, the experiment was conducted with native speakers of Chilean Spanish rather than British English, largely because Chilean Spanish is rhythmically distinct from English, as elaborated below (*Speech Data Pre-processing*). Second, whereas the original published data are mainly acoustic measures of alignment with occasional reference to movement visible on videos, the present study includes systematic motion capture of whole-body movement, thus allowing measures of motoric (bodily) alignment as well as speech alignment. Third, whereas participating dyads in the original studies were already friends, those in the present study did not know each other. Fourth, whereas the original studies were fairly naturalistic, the present study used similar tasks but in a more controlled experimental design, described below. The third and fourth innovations allowed us to track the development of interpersonal alignment in speech and movement rather than simply documenting its existence, and to assess whether an inherently pulsed rhythmic task (music) influences interactants’ speech alignment more than a cooperative but non-pulsed task.

We chose same-sex dyads who did not know one another at the beginning of the experiment and were not musicians, and we randomly assigned each dyad to one of two experimental groups. These two groups differed only in whether the non-conversational task a dyad took part in was to improvise music together, or to cooperatively build a tower using wooden blocks. Both tasks require cooperation, and a degree of manual skill that most people already possess in sufficient measure. Each also requires the hands to be busy, while eyes are often directed at the hands rather than the other person’s face. In previous work [49], we determined that pairs of people, whether with or without musical training, tended to organise their musical interactions around an emergent, shared pulse, entraining with one another in a process of mutual temporal co-adaptation [88]. Pulse-based joint behaviour is highly unlikely in the tower-building task, where the timing of the actions of each participant will depend on variable actions in the ongoing process of construction. Hence although both tasks—joint musical improvisation, and joint tower building—require coordination in time between participants, they differ substantively in their temporal features, with only musical interaction likely to be organised around a shared pulse. In sum, for each dyad, the experimental procedure began and ended with a short conversation and had an intervening task which was musical for some dyads and non-musical for others. Our measures compare alignment in body movement and periodicity of the speech in conversations 1 and 2, across the musical vs non-musical groups.

Our principal hypothesis, based on existing literature, is that musical interaction between non-musician interactants unknown to each other is likely to promote greater mutual behavioural alignment, both motoric and spoken, in subsequent conversational interaction than should a cooperative task that requires participants to sequence their movements cooperatively, but does not require them to maintain a shared pulse.

## Materials and methods

### Participants

Participants were invited to take part in a face-to-face conversation with an unknown participant as partner through advertisements distributed in the Pontificia Universidad Católica de Chile. Ninety-two undergraduate students volunteered for the study. None had had formal musical training or played an instrument. From this sample, 46 same-sex dyads of participants unknown to each other were generated and randomly assigned to one of two groups that differed in whether they took part in a joint musical improvisation (MI) or a ‘hands-busy’ task (HB), as explained in *Design* section below. This produced an initial set of 46 dyads, 26 in the MI group (15 female), and 20 dyads in the HB group (10 female).

The final group size was 13 dyads per group (seven females in MI, six in HB). 17 dyads (10 MI, 7 HB) were discarded due to loss of data (either Mocap or microphone malfunction). Three additional MI dyads were discarded because their adherence to the instructions to improvise music together (see Procedure below) was judged unsatisfactory. The selection was done by three of the authors, who independently watched videos of the MI interactions and ranked the musical interactions’ level of success on a 1–5 scale. The musical interactions typically took place as a series of fairly short bouts (as in [54]) interspersed with preparatory or reflective episodes, rather than extending continuously through the time allotted. Dyads were rated high (4 or 5) when participants paid attention to each other and played together for bouts of at least three seconds. Dyads were rated low (1–3) when they failed to engage with what the other was playing or played together for bouts of less than a few seconds. Kendall’s coefficient of concordance corrected for ties was computed and yielded a high inter-rater reliability (Wt = 0.738, chi-square = 55.4, p < .001). The average score was computed for each dyad, and the 13 highest-scoring dyads chosen for analysis, matching the number of selected HB dyads. The ratings of these 13 dyads were 3.3. or higher.

#### Motion capture

Participants’ motion was recorded with an optical motion-capture system consisting of 36 *NaturalPoint Prime-41* cameras and a computer running *Motive* optical motion capture software. This software package was used for calibration, 3D reconstruction, and motion capture recording. Cameras were installed close to the ceiling of the recording room, in a rectangular array above and surrounding the participants. Capture area was at least 3 meters x 4 meters x 2 meters (width, depth, height). Markers were located on the feet (2x), the knees (2x), the lower back (2x), the upper back (2x), the hands (2x), the elbows (2x), and the head (3x). Only the four back markers were analysed in the present data. Other markers were present to validate full body motion when visually inspecting recordings. The system used offers a practical compromise between naturalness and 3-D accuracy, sufficient for measuring alignment during dyadic interaction.

#### Audio and video recording

Each participant’s speech was recorded on separate channels using individual SHURE WH20 headset microphones and digitized through a TASCAM US 16x8 audio interface. Additionally, a Canon XF105 HD digital camera recorded each participant from in front.

### Design

The design involved a three-stage interaction: a conversation (T1), a joint activity that allowed but did not require conversation, and a second conversation (T2). The joint activity was either musical improvisation (group MI) or building a tall tower from wooden blocks, which kept the eyes and hands busy as in playing music but is a non-musical task that is likely to impose demands that render participants’ actions and interactions temporally irregular (group HB, for ‘hands busy’). Thus, the three-stage process was distinguished by what participants did between two conversations: playing music together (group MI) or cooperating in building a tower from blocks together (group HB).

### Procedure

Dyads were taken into the Language, Interaction and Phenomenology Laboratory (LIF) of the Pontificia Universidad Católica de Chile, where they read and signed an informed consent form previously reviewed and approved by the Ethical Committee of Social Sciences at the Pontificia Universidad Católica de Chile. Mocap markers and microphones were then placed as described above. Two low stools were placed opposite each other with a low table between them.

Once participants were seated, experimenters handed them a copy of the instructions and read them aloud. In both groups, the first and second conversations (T1 and T2) were free, but initially guided by an adaptation of the Fast Friends Questionnaire [89] for inducing talk among students who are strangers and meeting for the first time. Conversations consisted of a series of mutual questions about participants’ names, studies, etc. After T1, group MI improvised music together, while group HB built a tower together using wooden blocks. The HB participants were told “*les vamos a pedir que construyan juntos una torre y que la pasen bien”* [we’ll now ask you to build a tower together and have fun]. For group MI, a variety of instruments (metallic and wooden xylophones, several small drums and a tambourine) were placed within easy reach on side tables next to each participant. One of the most salient aspects of spontaneous musical interaction is its organisation around a shared pulse or beat [49]. Such pulse-sharing is called “entrainment”, in the sense intended by Clayton, Sager & Will [41]. To facilitate entrainment, the instruments available to participants were all percussive (specifically, struck idiophones), expediting the articulation of rhythm and pulse. It should be noted that although the participants were not instructed to behave rhythmically, when they managed to engage musically with each other during the experimental sessions they tended spontaneously to organise their musical behaviours around a regular beat.

Because participants had essentially no music performance experience, each MI dyad was initially familiarized with the instruments, being instructed to “*les vamos a pedir que*, *juntos*, *la pasen bien explorando los instrumentos que ven sobre esta mesa*” [have fun together exploring the instruments you see on this table]. Familiarization lasted an average of 2’19” (2’10” to 2’43”). As Hawkins, Cross & Ogden [54: 321–3] had found that the specific instructions given to participants substantially affected the extent to which participants (same-sex friends) engaged cooperatively and successfully in their musical task, we carried out a series of pilot studies in order to develop appropriate instructions for our participants, who were all same-sex strangers. In the pilots we noted a tendency for participants to engage individually with the musical instruments or in talk about music rather than make music together; we tested a range of possible instructions that could help break the ice, and found that asking them to play some sad and then some happy music together was most successful in eliciting joint musical behaviour. Accordingly, participants were told “les vamos a pedir que, entre los dos, toquen algo que suene triste” [play, together, something that sounds sad] and after that “les vamos a pedir que, entre los dos, toquen algo que suene alegre” [play, together, something that sounds happy]. Our intention was neither to induce happiness or sadness in the participants nor to impose particular aesthetic qualities on their interactions, but simply to help them to make music *together*. As in [54], we aimed to be as explicit as was required for the interaction to be cooperative and naturalistic while avoiding over-prescriptiveness by providing them with an easily understood common goal.

Mean durations (and ranges) of tasks were as follows: Initial conversation, T1: group MI, 1’53” (1’01” to 4’10”); HB group, 2’16” (1’16” to 3’53”). HB task: 5’36” (2’38”to 8’36”). Musical improvisation: 5’51” (4’51” to 7’57”). Second conversation, T2: MI group 5’37” (3’10” to 11’05”); HB group 9’13” (3’51” to 18’14”). Finally, each participant was paid the equivalent to 8 USD and their questions were answered before they left the lab.

### Analysis

#### Motion capture (Mocap) data

*Mocap data pre-processing*. The conversation Mocap data (T1 and T2) was exported and then each marker was trajectorized over time. Next, we manually labelled markers with their corresponding body parts and identified the participant to which each marker belonged. Finally, we visually inspected the results to identify where data for a marker had been lost. Such gaps were filled by linear interpolation.

*Computation of speed cross-correlation curves*. Our data comprised position x time series pairs, corresponding to the period between 0 s and 60 s. Each pair consisted of one time series for the positions of one participant and another one for the other participant. Following Cornejo et al. [62], we first averaged back markers for each participant into a single 3D position and only kept 1D positions of the proximity axis (i.e., between the two subjects). Second, we computed discrete speed signals (distance over time within each measurement period) by subtracting each marker’s proximity (1D) position at each frame from its position in the immediately previous frame. Third, a single pole 10-Hz cut-off frequency low-pass filter was applied to speed signals in order to exclude the possibility of fast recording artifacts, since typical human motion does not exceed 10 Hz [90]. Fourth, for each pair of time series, we visualized synchronous and delayed coordination patterns by computing a Pearson cross-correlation curve for temporal offsets between -1.5 s and +1.5 s of the referent, discarding signal ends that did not overlap. This computation normalizes the scale by constraining values to between -1 and +1.

Cross-correlation curves from the same experimental condition were aggregated by pooling rather than averaging, with confidence intervals calculated by the Fisher transform for p-values. A Bonferroni correction for the 31 values in each cross-correlation curve was applied: alpha level for confidence interval visualisation was 0.0001. The result was a single cross-correlation curve (with confidence intervals) for each condition, whose values can be statistically tested for significance of differences. These are plotted with the Pearson correlation on the vertical axis and time delays relative to the referent on the horizontal. A high magnitude correlation at t = 0 represents a tendency for couples to move in a similar fashion and at the same time.

### Speech data pre-processing

#### Identification of pike location in Chilean Spanish

Loehr’s [85] work on American English, confirmed for British English by Ogden and Hawkins [54], indicates that a very reliable acoustic property associated with gestural pikes is an f0 maximum or minimum on syllables carrying pitch accent, or primary stress. Although Chilean Spanish uses a number of distinctive f0 patterns [91, 92] and has accented syllables (“sílaba tónica” being the main stress in a citation-form (isolated) word), by far the most common f0 pattern within an accented syllable is monotonic. That is, accented syllables typically have no f0 peak in Chilean Spanish. As there is no agreed acoustic-phonetic criterion for accentuation in Chilean Spanish, our first task was to develop one. We wanted it to be as comparable as possible to the criterion used for English. We used the intensity maximum for two reasons. First, intensity has long been widely accepted as related to perceived syllable prominence in English, and (other things equal) often varies fairly closely with f0—see [93]. Second, maxima in both f0 and intensity curves are reliably measurable, and any difficulties or ambiguities tend to be of a similar nature (such as very late maxima, or prolonged plateaux), and so are resolvable by judgements that rely on similar expertise. In short, a display of the intensity curve allowed a single point to be located in each Chilean accented syllable, as an operational implementation of Loehr’s concept of pike, about as satisfactorily as f0 maxima/minima in accented English syllables with pitch accent.

#### Q+A extraction and pike identification

**Fig 1:**
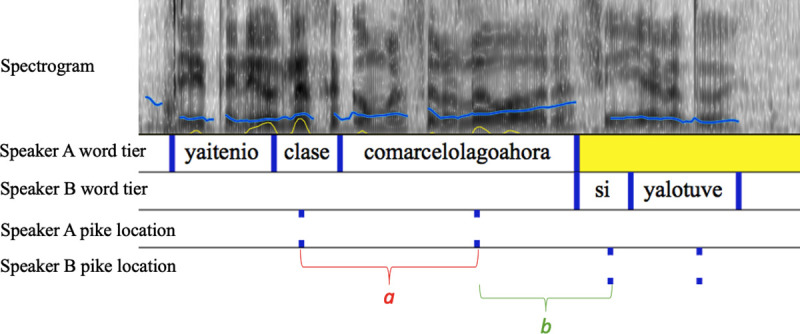
Screenshot of a Q+A pair set up in Praat. Top panel: spectrogram of both talkers’ speech (0–500 Hz), with speakers’ f0 contour (blue) and intensity contour (yellow) superimposed. Next two panels: speaker A and speaker B’s words, on separate tiers. (The compressed time scale means that the word *abuela* has been truncated to *abue*.) Lowest two panels: speaker A’s and speaker B’s pikes. The red letters *a*, *b*, *c* and *d* at the bottom of the figure denote the four (pike-to-pike) time intervals between the five pikes in the Q. The green letter *e* denotes the interval between the last pike of the Q and the first one in the A (across the turn space). The total duration of the speech displayed is 2.9 s.

226 spontaneous Q+A pairs were extracted from the data using Audacity v 2.1.2 [94]. These comprised all Q+A pairs except those read aloud from the Fast Friends cards. Two trained phoneticians, native speakers of Chilean Spanish, independently set up text grids in Praat [95] with each talker’s words in separate interval tiers. Accented syllables were identified by listening, and pike location within each accented syllable was located from the displayed intensity curve and represented in point tiers (Fig 1).

This initial corpus was analysed as follows. First, the two Chilean phoneticians worked independently. They then combined their work, distinguishing Q+A pairs that they agreed on independently from those that they agreed after discussion, and from those they could not agree. Then the first author, also a native speaker of Chilean Spanish but not a trained phonetician, checked the phoneticians’ work. All disagreements were referred to the second author, a phonetician and English native speaker, who examined them independently. These two authors then made a decision about each disagreed pike, and sent their conclusions to the two Chilean phoneticians, separately. Pike locations were accepted when both Chilean phoneticians independently agreed with the authors’ decision.

The 226 Q+A pairs were fairly evenly divided between the two groups: 118 and 108 for MI and HB respectively. Table 1 shows that, for the 882 pikes identified in these 226 Q+A pairs, 812 were agreed by all judges (420 and 392 for MI and HB groups respectively). Of these, 787 were agreed independently by the Chilean phoneticians and the first author. The remaining 25 were instances in which the initial analysis of the structure was revised e.g., a single Q was reclassed as a sequence of two Qs.

**Table 1:** Number and percentage of agreements and disagreements about pike location per condition (MI and HB) and overall (total) in the initial corpus.

	Pikes in the original 226 Q+A pairs				
	Agreed		Disagreed		Total
Group	Number	% for condition	Number	% for condition	Number
MI	420	93	32	7	452
HB	392	91	38	9	430
Total	812	92	70	8	882

The remaining 70 disagreements on pike location were distributed across 82 Q+A pairs. Most disagreements (61, 87%) concerned the syllable on which a pike was placed. Usually it was either omitted by one or more judges or placed on an adjacent syllable. Some instances concerned whether clicks, in-breaths, and the Spanish equivalent of *um*, *er* that presaged an A entry were classed as pikes by the Chilean phoneticians. In the other 9 cases (13%), there was agreement on the syllable, but disagreement on the exact location of its pike, with an average discrepancy of 80 ms.

When a disagreement about pike location could not be resolved, its Q+A pair was rejected. The final corpus comprised 195 Q+A pairs, 91 from group MI, 104 from HB. These data are returned to in the Results, Table 2.

**Table 2:** Descriptive statistics for the spontaneous Q+A pairs produced by MI and HB groups in the final dataset, in T1 and T2 conversations.

		Q+A pairs (frequencies)		Durations of conversations (s)	
Measure	Group	T1	T2	T1	T2
total n	MI	30	61		
	HB	29	75		
mean (SD) per dyad	MI	2.31 (1.59)	4.62 (2.06)	113 (36)	337 (133)
	HB	2.30 (2.00)	6.50 (5.73)	136 (30)	553 (292)
median (range) per dyad	MI	2 (0–4)	5 (1–9)	110 (61–250)	332 (190–665)
	HB	2 (0–7)	4 (1–22)	136 (76–233)	418 (231–1094)

#### Computation of periodicity in Q+A pairs

The next step was to classify the Q+A pairs in terms of their periodicity. Ogden and Hawkins [54] first designated the Q as periodic or aperiodic (though as noted above they used the terms rhythmic and arhythmic). Then, for periodic Qs only, they assessed whether the first pike of the A entered the turn space with the same periodicity as that set up by the Q. They defined periodic (rhythmic) Qs as those whose intervals between successive pikes had the same duration ±15%. This criterion was applied sequentially across all adjacent pike intervals to assess a Q’s periodicity.

Our methods were closely related, but to allow us to more thoroughly explore types of relationship between periodicity of the Q and that of the A’s entry into the turn space, we used three different estimates of periodicity in the Q, always with the same ±15% criterion. The name of each measurement method reflects what it measures in the Q.

1. *1interval*: Sometimes called *last interval* (see below). The duration between the last two pikes in the Q (regardless of the degree of periodicity in any potential earlier pike-to-pike intervals) and the duration between the last pike in the Q and the first pike in the A. When the interval across the turn space differed by no more than ±15% from that of the single interval identified in the Q, the Q+A pair was classed as ‘Periodic (1interval)’. This 1interval measure is used in two mutually-exclusive contexts, referred to by different terms that indicate the type of question the measurement is applied to. (a) 1interval: used when there are only two pikes in the Q, so it has only one pike-to-pike interval. Such questions are called single-interval Qs. (b) last interval: when the Q contains multiple pike-to-pike intervals (called a multi-interval Q), but the periodicity measurement assesses only the last of these.
2. *2interval*: When the last two pike-to-pike intervals of a multi-interval question differed by no more than ±15%, then the end of the Q was ‘Periodic’. If the interval across the turn space following such a periodic Q differed by no more than ±15% from the last interval in the Q, then the Q+A pair was termed ‘Periodic (2interval)’. This is in large measure the same criterion as Ogden and Hawkins [53] used for their ‘Rhythmic’ category. (Although Ogden and Hawkins did not restrict the number of pikes measured in the Q, in practice, most of their Qs had only 3 or 4 pikes, and so only 2 or 3 pike-to-pike intervals.)
3. *AverageInterval*: When the interval across the turn space differed by no more than ±15% from the average duration of a multi-interval Q’s pike-to-pike intervals, then the Q+A pair was termed ‘Periodic (AverageInterval)’.

These relationships are illustrated in Fig 1 and expressed mathematically below. Speaker A asked a multi-interval Question with 5 pikes, shown in the “Speaker A pike location” tier of Fig 1. Speaker B answered it. Only one pike is notated for speaker B’s answer (bottom tier of Fig 1), since no others are used in the measurement. In Fig 1, intervals between two pikes in the Q are labelled *a–d* in chronological order (in red). The interval across the turn space is labelled *e* (in green). For example, intervals *d* and *e* are used to calculate the *last interval* measure of periodicity for this multi-interval Q. Their durations are 412 ms and 283 ms respectively. Since neither 412 nor its half-cycle, 206, are within 15% of 283, the Q+A pair is classed as Aperiodic by the last interval criterion.

The following formulae show the calculations for *1interval*, *2interval* and *AverageInterval*:

$$1interval:\;f(d,e)=\left\{ \begin{array}{c} 1,\;ifd/e\in [.85,1.15]\\ 0,\;\mathrm{otherwise} \end{array}\right.$$ (1)

$$2interval:\;f(c,d,e)=\left\{ \begin{array}{c} 1,ifc/d\in [.85,1.15]\wedge d/e\in [.85,1.15]\\ 0,\;\mathrm{otherwise} \end{array}\right.$$ (2)

$$AverageInterval:\;f(x_{1},x_{2},\ldots ,x_{n},e)=\left\{ \begin{array}{c} 1,\;\mathrm{if}\frac{\sum_{i=1}^{n}x_{i}}{ne}\in [.85,1.15]\\ 0,\;\mathrm{otherwise} \end{array}\right.$$ (3)

The elements *a*, *b*, *c*, *d* refer to intervals between pikes in the Q, as explained above and shown in Fig 1. Likewise, the element *e* refers to the interval across the turn. In each case, 1 refers to a ‘Periodic’ classification of the Q+A pair, and 0 to an Aperiodic classification. (Consistent with standard notation, in the *AverageInterval* formula, *n* = the number of pike-to-pike intervals in the Q, and *x*_*i*_ to any such interval.)

Each of these measures was applied to each Q+A pair, except that only the 1interval measure can be applied to single-interval Qs, as explained above.

## Results

### Motion capture data

**Fig 2:**
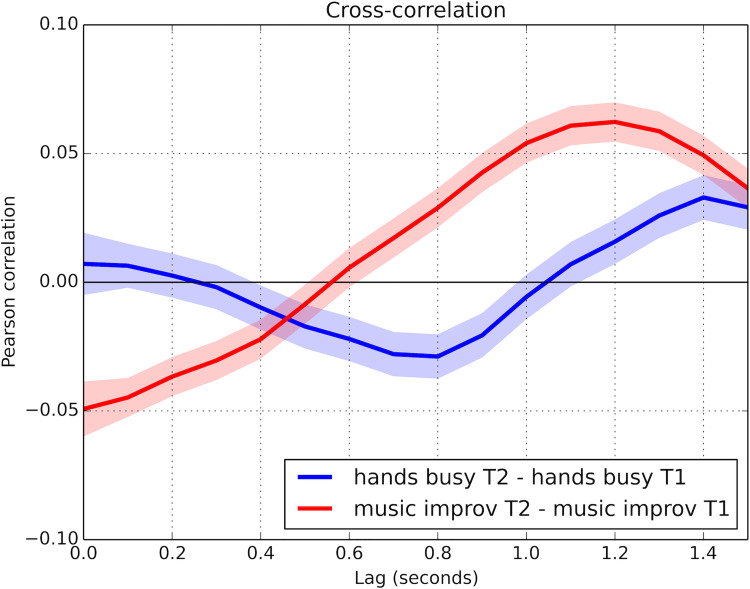
Cross-correlation curves showing the subtraction of the post-activity coordination (T2 correlations) from the pre-activity coordination (T1 correlations) for both groups. Shading represents Bonferroni-corrected 99.99% confidence intervals. A statistically significant difference of p < 0.0001 is implied when shaded areas of two curves do not touch or overlap at a given time.

Fig 2 shows the subtraction of T2 correlations from the initial T1 correlations. The amplitudes give an indication of the differential impact of the activity (hands-busy vs musical improvisation) on the coordination observed during the initial conversation in both groups. Curve amplitude indicates the magnitude of the bodily coordination between interactants. Positive correlations evidence mirror coordination (i.e., when A moves forward, B moves forward); negative correlations indicate anatomical coordination (when A moves forward, B moves back). The plot displays the correlations along the time from 0 s (i.e., simultaneous coordination) to 1.5 s (i.e., delayed coordination). To visualise variability, the shading around each curve represents the Bonferroni-corrected 99.99% confidence interval around computed correlation values (alpha = 0.0001). The difference between pre- and post-activity coordination in the MI condition shows peaks at 0s and at *ca*. 1.1–1.2 s, indicating a tight temporal coupling between participants’ movements while conversing after the musical interaction; this is not apparent in the HB condition.

**Fig 3:**
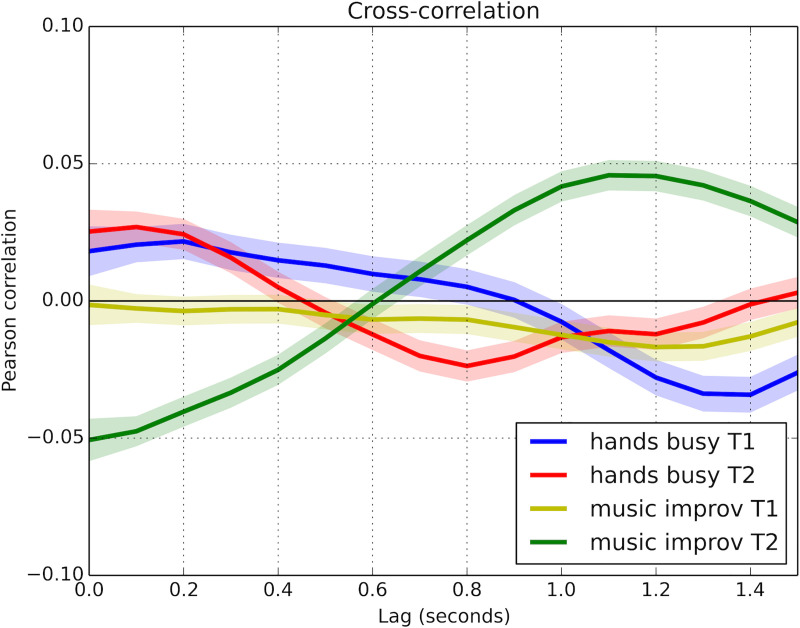
Aggregated cross-correlation curves for hands-busy and music improvisation groups in two different conversation moments: Before (T1) and after (T2) the joint activity. Shading represents Bonferroni-corrected 99.99% confidence intervals. A statistically significant difference of p < 0.0001 is implied when shaded areas of two curves do not touch or overlap at a given time.

Fig 3 shows the magnitudes of coordination between the participants during the conversations held before and after the intermediate activity for both groups. The HB-T1 curve (blue) represents the degree of coordination between interactants when chatting before the hands-busy activity, while the HB-T2 curve (red) shows the coordination between them when talking after the hands-busy activity. Likewise, MI-T1 (yellow curve) and MI-T2 (green curve) chart, respectively, the coordination between participants when chatting before and after the musical improvisation activity.

Fig 3 shows that M1-T2 has a distinctive, simple trajectory compared with the other three curves. Moreover, it is the only curve with a negative correlation at zero lag (r = 0.05, p<0.001), and it remains negative and significantly different from that of MI-T1 (confidence intervals do not overlap) for almost all of the first half second of data. In contrast, confidence intervals for the non-significant positive correlations of HB-T1 and T2 overlap considerably during this period. This result indicates anatomical coordination between participants in the window 0 s—0.4 s during the final conversation only after the musical improvisation activity. Furthermore, MI-T2 curve exhibits a relatively high positive amplitude in the window 0.8 s—1.5 s (highest peak at 1.1 s lag) and its morphology reveals mirroring coordination. This pattern contrasts with the other three curves, which present negative values at the same lag. Thus, in the musical improvisation condition, curve shape indicates a significant variation from the initial conversation (MI-T1), where coordination is practically non-existent, to the conversation after the musical activity, where coordination is the most expressive. Fig 3 also evidences small differences between coordination at T1 and T2 in the hands-busy condition; both curves show delayed coordination (indicating a degree of imitation), although the lag is shorter in T2 (peak 0.8 s lag) than T1 (1.3 s lag).

### Speech data

#### Distribution of Q+A pairs

The left hand side of Table 2 shows descriptive statistics for the Q+A pairs, for each group and conversation. The right hand side of the table shows equivalent durational data for the conversations. Column 3 shows that, in T1, the total number of Q+A pairs, and the average and median per dyad, were almost identical for the MI and HB groups. Both groups produced more Q+A pairs during T2 (Column 4). Superficially, the difference is partly explained by the longer duration of conversations in T2 than T1 (Columns 5 and 6 in Table 2). However, Pearson correlations between the number of Q+A pairs and the duration of the conversation were only significant for MI in T2 (r = .80, p = 0.001). Correlations were non-significant for HB in both T1 and T2, and for MI in T1: r = .45, p = 0.124; r = .56, p = 0.073; and r = .41, p = 0.164 respectively. This pattern suggests that, with the exception of MI T2, the number of Q+A pairs produced by dyads in a particular conversation cannot be interpreted as a direct result of conversational duration.

Table 3 shows the number of single-interval and multi-interval Qs in each group, together with totals for both the groups and the type of Q. The two groups differed little in the total number of questions asked and answered, but a χ^2^ test of association confirms that MI asked more single-interval and fewer multi-interval Qs than expected by chance, with HB tending in the opposite direction (Fisher’s exact test, 2-tailed p = 0.031).

**Table 3:** Number of single- and multi-interval Qs, per group.

Group	single-interval Qs	multi-interval Qs	Total
MI	55	36	91
HB	46	58	104
Total	101	94	195

#### Periodicity of Q+A pairs

Periodicity analyses were performed using the three measures described in the subsection *Computation of periodicity in Q+A pairs*. Of the total 195 spontaneous Q+A pairs analysed, 78 (40%) were classed as having Periodic answers by at least one of the three criteria. A score of 1 was assigned to Q+A pairs that had periodic answers, and 0 to pairs with aperiodic answers. A number of analyses were conducted on various parts of these binary periodicity data. For each, we fitted a logistic regression model, with Group (HB or MI) as a between subject factor, Conversation (T1 or T2) as a within-subject factor, and Dyad as a random factor. All analyses were conducted in R (Version 4.0.2, R Development Core Team) using the *lme4* package [96] with an ANOVA using a Type III Wald chi-square Analysis of Deviance test applied to the model output. Critical alpha level for significance was p = 0.05. All t-tests are two-tailed.

**Fig 4:**
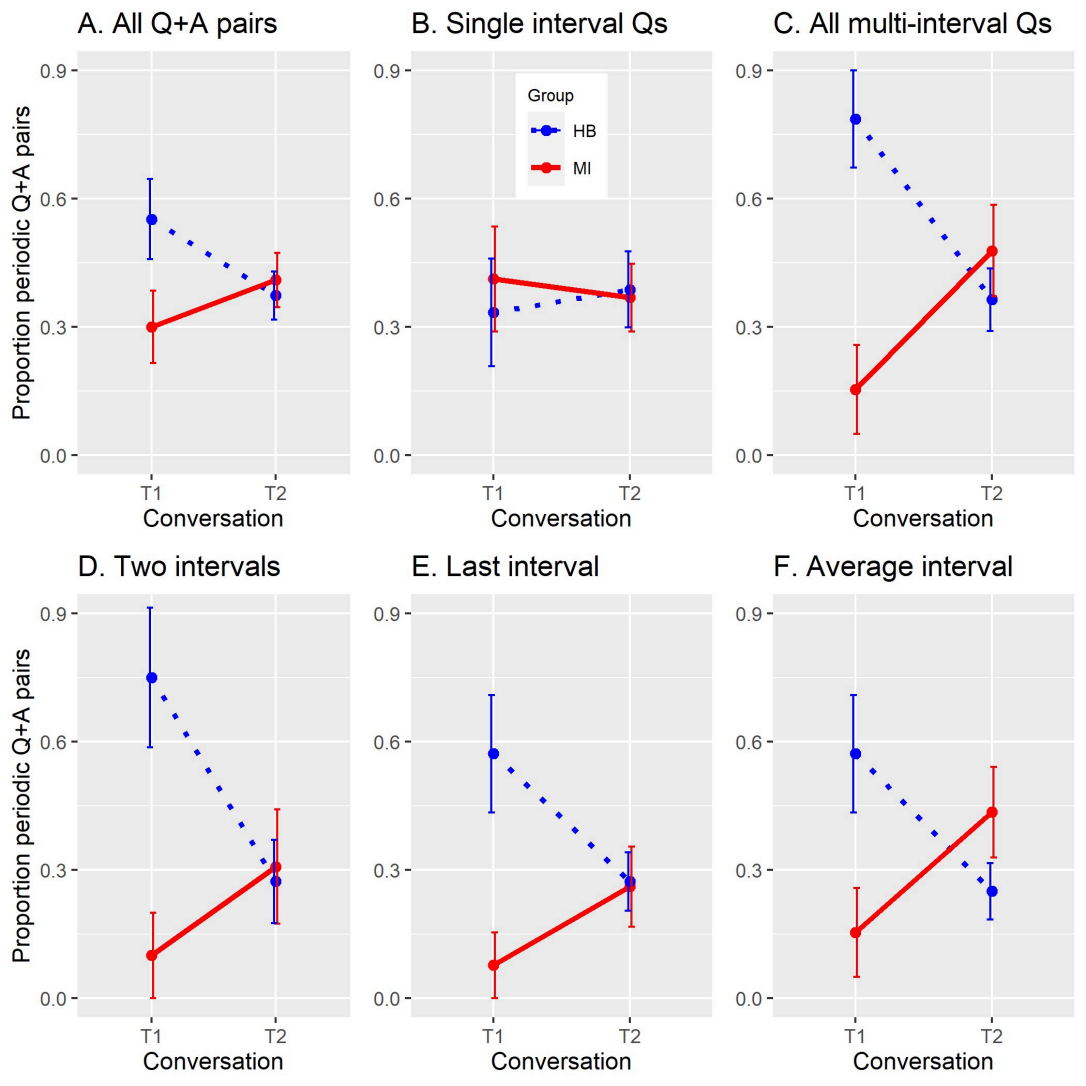
Proportion of periodic Q+A pairs per group (MI, HB) and conversation (T1, T2). A: total Q+A pairs. B: single-interval Q+A pairs only (1interval measure). C: all multi-interval Q+A pairs, on all three measures. Bottom row: Multi-interval pairs, for each of the three different measures. D: 2interval. E: last interval (using 1interval measure). F: AverageInterval. Line graphs are used to emphasize the effect of interest. Group MI: solid red. Group HB: dotted blue. Error bars: standard error.

The first analysis was on the complete set of 195 Q+A pairs—that is, single interval and multi-interval pairs together. These data are shown in Fig 4A, in terms of proportion of periodic Q+A pairs relative to the total number of Q+A pairs. The main effect of Conversation was not significant [χ^2^(1) = 2.68, p = 0.1], while both Group and Group x Conversation were marginally significant: Group [χ^2^(1) = 3.73, p = 0.053], Group x Conversation [χ^2^(1) = 3.45, p = 0.063].

The data were then subdivided and analysed separately according to the number of intervals in the Q. These data are shown in Fig 4B and 4C, for single-interval and multi-interval Q+A pairs respectively. Of the 101 single-interval Q+A pairs, in which all Qs had just one beat, 38 were classed as periodic by the 1interval measure (the only measure applicable to single-interval Qs). Fig 4B shows no difference between the groups and conversations for these single-interval data (Group [χ^2^(1) = 0.21, p = 0.65], Conversation [χ^2^(1) = 0.19, p = 0.66], Interaction [χ^2^(1) = 0.22, p = 0.64]). Fig 4C, in contrast, shows the pattern of Fig 4A in amplified form, confirming that the main influence seen for the interaction pattern in the complete data set comes from the multi-interval Q+A pairs. The single-interval pairs exert no influence on the dominant pattern.

For the model fit to the 94 multi-interval Q+A pairs, 40 (43%) of which were classed as Periodic by at least one of the three classification measures, each main effect and their interaction were significant: Group [χ^2^(1) = 8.89, p = 0.003], Conversation [χ^2^(1) = 7.24, p = 0.007], Group x Conversation [χ^2^(1) = 9.39, p = 0.002] (the analysis only counted each pair once) Although the two groups appear to show opposite patterns (Fig 4C), the only significant simple main effect in this interaction was that for Group at T1 (unpaired t(24) = -2.256, two-tailed p = 0.03, Cohen’s d (using MI variance) = 0.990). On the main parameter of interest, MI’s predicted rise in periodic Q+A pairs is observed, but neither that, nor HB’s fall, reaches statistical significance. (MI: paired t(12) = -1.642, p = 0.1, Cohen’s d (using pooled variance) = 0.489. HB: t(12) = -1.091, p = 0.2, Cohen’s d (using pooled variance) = 0.351. (The binary data used for the logistic regression do not allow simple main effects to be calculated. To perform these and later t-tests, we converted the data to a by-dyads analysis (26 items per group, 13 T1 and 13 T2 for each group) where each item represented the percentage of periodic pairs relative to that specific dyad’s total number of pairs.)

The above analyses of the multi-interval Q+A pairs lump together periodicity counts from all three measurement criteria (2interval, last interval, and AverageInterval). The same analyses were also run separately for each measure. For last interval and AverageInterval measures, proportions are again calculated relative to the full set of 94 multi-interval Q+A pairs, since both measures are applicable to all multi-interval pairs. For the more stringent 2interval measure, proportions of periodic Q+A pairs are calculated relative to the 53 questions in which the last two pike-to-pike intervals were within ±15% of each other. The bottom row of Fig 4 shows similar patterns of data for each of the three measures, with the strongest interaction for the pairs classified as periodic by the AverageInterval measure (Fig 4F). Furthermore, while effects of Group and the Group x Conversation interaction were significant for all three subsets (Fig 4D–4F), the main effect of Conversation (T1 vs T2) was significant only for the AverageInterval data. Details are as follows. 2interval: Group χ^2^(1) = 5.94, p = 0.01; Conversation χ^2^(1) = 3.47, p = 0.06; Group x Conversation χ^2^(1) = 5.07, p = 0.02. Last interval (1interval measure): Group χ^2^(1) = 5.15, p = 0.023; Conversation χ^2^(1) = 3.06, p = 0.08; Group x Conversation χ^2^(1) = 4.29, p = 0.038. AverageInterval: Group χ^2^(1) = 6.07, p = 0.014; Conversation χ^2^(1) = 5.24, p = 0.022; Group x Conversation χ^2^(1) = 6.78, p = 0.009.

The significant effect of Conversation in the AverageInterval measure encouraged us to examine that subset of data in more detail. As described above, the average-interval data were converted to a by-dyads matrix (with independent variables Group and Conversation, the dependent variable being each dyad’s percentage of periodic pairs relative to that dyad’s total pairs). As with the total data, the difference between groups at T1 remains significant (HB > MI, Welch two-sample t(14.08) = 2.25, p = 0.041), and the decline for HB between T1 and T2 is not significant (HB, T1 > T2, paired t(12) = 0.93, p = 0.37). However, in contrast with all other measures, the predicted increase for MI results is non-significant: MI, T2 > T1, paired t(12) = -2.16, p = 0.052.

We also asked to what extent the three measures classified the same Q+A pairs as periodic, and where any differences lay. Table 4 shows that 13 pairs were classed as periodic by all three measurement criteria, and that the AverageInterval measure most often classed Q+A pairs as periodic when the other two measures did not (13 pairs, Exclusive column, Table 4). In contrast, only 5 Q+A pairs qualified as periodic on the last interval criterion alone (with another 4 qualifying on both last interval and 2interval criteria but not AverageInterval). Interestingly, no Q+A pairs were classed as periodic on the 2interval criterion alone, although, as noted, 4 qualified on 2interval as well as last interval, and 13 on all three criteria. That is, if a Q+A pair qualified as periodic on the 2interval criterion, it also qualified as periodic on either the last interval criterion, or all three criteria, but never with AverageInterval alone. This pattern probably simply reflects the greater stringency of the 2interval criterion compared with the other two. Overall, the implication is that the AverageInterval measure is the most reliable single indicator of Q+A periodicity in this study.

**Table 4:** Number of multi-interval Q+A pairs classed as periodic, by type of measure.

		Number of periodic classifications					
	Measure	Exclusive	Last int+2int	Last int+AvInt	2int+AvInt	All three	Total
Measure	Last int	5	4	5	na	13	27
	2int	0		na	0		17
	AvInt	13	na	5			31
	Total	40					

Each row represents a given measure. Column 3 (Exclusive): number of Q+A pairs classed as Periodic by the criterion listed in the row label ‘Measure’. Last int+2int: number of pairs classed as Periodic by both last interval and 2interval measures but not the AverageInterval measure. Similarly, for the other columns (‘All three’ means last interval, 2interval and AverageInterval criteria).

Finally, we asked whether periodicity was associated with the timing of answer entries. Following [54, 86], a periodic-entry Answer is classed as on-time if its first pike occurs on the next beat after the last pike in the Q; as late if its first pike comes in on-beat but after the beat that follows the last pike of the Q; and as early if it is on-beat, but occurs with or before the last pike of the Q. In our 40 multi-interval periodic Q+A pairs, 11 (28%) Answer entries were On-time, 24 (60%) were Late, and 5 (13%) Early. This pattern resembles that found by Ogden & Hawkins [54] for pairs of friends. There were no significant effects for early- and on-time entries together (pooled to avoid low n): Group [χ^2^(1) = 0.00, p = 0.98]; Conversation [χ^2^(1) = 0.00, p = 0.93]; Group x Conversation [χ^2^(1) = 0.00, p = 0.98]. However, for late-entry Q+A pairs, statistically significant effects were found for Group [χ^2^(1) = 5.09, p = 0.02], Conversation [χ^2^(1) = 6.48, p = 0.01] and their interaction [χ ^2^(1) = 6.97, p = 0.01]. Numbers were more evenly distributed for the single-interval pairs: 15 on-time and 19 late. This presumably means that single-interval questions, which by definition are short, tend to be easier to answer.

## Discussion

The Introduction identified three general questions. All have been answered affirmatively. The periodicity across conversational turns observed by Ogden and Hawkins [54] for English is also found for (1) conversants speaking Chilean Spanish, a language whose prosody differs markedly from that of English, (2) when they do not know each other. (The influence of familiarity of interlocutors is reflected in the overall incidence of periodicity: about 40% and 70% for the present study and Ogden & Hawkins [54], respectively.) (3) The nature of the particular ‘joint-action’ task—by hypothesis its inherent rhythmicity—does appear to affect results. The following sections consider the details, first for motion capture and speech separately, and then in relation to each other.

### Motion capture and speech results

Motoric data showed a significant increase in coordination in the MI group after jointly improvising. Compared to the HB group, during the second conversation (T2), participants in the musical group evidenced: (a) very rapid, partially simultaneous anatomical coordination in the 0–0.4 s time window; and (b) slightly delayed mirror coordination between 0.8 and 1.5 s. The body movements of both listener and speaker entrained to each other to a greater extent than evidenced in the HB group, first anatomically—when participant A moves forwards, participant B moves backwards, and then in a mirror-like fashion—when A moves forwards, B moves forwards too. That is, simultaneous body coordination occurred only after the musical improvisation activity and not after the non-periodic cooperative task. In terms of temporal unfolding, these findings indicate that interpersonal coordination is significantly tighter after a musical activity than after a cooperative, but non-periodic one. In general terms, these results confirm that musical interaction produces a greater increase in gross interpersonal motor alignment at the level of the interlocutors’ trunks.

In their speech, the two groups produced similar numbers of Q+A pairs, especially in the first conversation (T1), but differed in the proportions of multi-interval periodic pairs across the two conversations, resulting in the significant Group x Conversation interaction shown in Fig 4: the MI group produced a higher proportion of periodic multi-interval Q+A pairs during the second conversation (T2) compared with the first (T1), whereas the HB group produced rather fewer. Taken by itself, this pattern between T1 and T2 suggests that musical improvisation weakly facilitated subsequent spoken coordination.

However, the Group x Conversation interaction depends most strongly on the fact that the HB group produced a higher proportion of periodic Q+A pairs than the MI group during the first conversation (T1), yet much more similar proportions in T2. The scope of the present study does not allow us to conclusively explain this difference in T1, but it does allow a unified tentative explanation that could be tested in later work. The explanation rests on the pragmatic function of answers to questions, and how these may have differed between the groups, due to the differences between the two experimental conditions.

As noted in the Introduction, Conversation Analysis attributes periodic (on-beat) entries of answers into a turn space to so-called preferred answers. Aperiodic (off-beat) answers are typically, though not inevitably, attributed to dispreferred answers [54, 86]. Both types of answer can represent skilful conduct of a successful conversation. Preferred answers allow expression and perception of shared intentionality. They tend to be relatively straightforward, with tight timing and convergence of other linguistic and phonetic parameters. Dispreferred answers can indicate some sort of reservation—hedging (*yes but…*), or disagreement with an implied presupposition, which makes the answer more complicated. So, an aperiodic answer (non-rhythmic entry into turn space) can facilitate communication because its pragmatic function is to herald some complexity which makes a simple response unlikely.

Our speech data indicate no difference between the two groups in adherence to the instructions—they produced very similar absolute numbers of Q+A pairs—but for Group MI, the preponderance of short (single-interval) Qs, together with changes to the percentage of periodic Q+A pairs, suggest strong differences from HB in the pragmatic functions of the answers offered to the questions asked, and a tendency for these functions to change between the first and second conversations. That Late entry on-beat Answers contributed more than Early- and On-Time Entry answers to the statistical interaction further supports the interpretation that pragmatic factors played an important role in the pattern of the present data. We can therefore infer both a difference in the starting state of the two groups which was unlikely to have arisen by chance, and differences introduced by the experimental manipulation (MI vs HB).

A tentative, yet parsimonious, explanation is as follows. The MI group were aware from the outset that they were likely to be asked to play the instruments. This awareness may have induced during T1 anxiety in some participants, and/or interest in the instruments themselves. Both reactions seem likely to introduce a degree of inattention paid to the simple types of questions they were asking (e.g., *What year are you*? *What’s your major*? *Are you from Santiago*? *Do you live with your parents*? *Do you practice a sport*? *What courses are you taking*? *What kind of music do you like*?). Either reaction could result in a majority of aperiodic multi-interval Q+A pairs in T1, while producing no difference in the single-interval questions, which, arguably, might demand less attention.

In contrast, the HB group seems less likely to have been anxious about the upcoming tower building task, nor to have been so distracted by the inherent interest afforded by wooden blocks. The questions asked in T1 typically have simple (preferred) answers, so we infer that the HB group seems likely to have produced a higher proportion of periodic Q+A pairs than the MI group, because they were probably attending more closely to the conversation itself in T1.

In summary, we suggest that the distribution of periodic Q+A pairs between the two groups and the two conversations probably reflects differences in the attention paid to them by the person answering. The next section develops this potential explanation, while considering the motoric alignment data too.

### Inter-domain convergence

As a whole, motor and speech data converge in confirming that joint music-making—a mainly non-verbal, pulsed collaborative task—promotes interpersonal temporal alignment in a subsequent speech task. Joint music-making not only tended to increase speech alignment as represented by Periodic Q+A pairs, but also generated stronger mirror-like bodily coordination when compared with the affiliative but non-periodic hands-busy task.

The MI results indicate a number of differences from the HB group. These are noteworthy since both groups’ conditions were structured so as to promote affiliative attitudes and collaborative engagement in a fairly relaxed brief interaction. Both were game-like, non-competitive forms of joint action. However, improvising music together seems to promote physical interpersonal coordination after the music-making has stopped more than the hands-busy task did. The difference between the two tasks suggests that the entrainment that emerges during musical improvisation facilitates the formation of more coordinated, talker-sensitive interaction.

Notwithstanding the fact that the MI group shows greater differences between T1 and T2 in motoric coordination, cross-correlation curves corresponding to the first conversation (T1) are not identical across experimental groups as one might have expected. Indeed, MI dyads evidenced particularly low baseline (T1) cross-correlations. Likewise, the proportion of Periodic Q+A pairs in MI T1 is also lower when compared to T1 in the HB group. The most parsimonious explanation for these initial dissimilarities can be found in the operationalisation of our experiment. While assignment to group was random, during the initial conversation participants in the musical improvisation group were likely to have inferred, from the presence of musical instruments in the experimental setting, that they would be asked to perform a music-related task. The literature suggests that imminent music-making can induce a significant degree of performance anxiety in ‘non-musicians’ [97–99], so such anxiety may well have interfered in their interpersonal alignment.

An alternative, though related, explanation for the higher levels of coordination in the MI group in the second conversation session could be that participants experienced release from stress on having successfully negotiated a task that they had perceived as difficult or socially awkward prior to tackling it [98], resulting in positive and shared affective states. This could have led to enhanced coordination and a greater sense of mutual affiliation, manifested in higher levels of interpersonal coordination. It could also be the case that the positive and shared affective states arose simply by virtue of having been rhythmically entrained in the course of the musical interaction; several studies have shown that shared rhythm is directly associated with—indeed, entangled with—a degree of emotional alignment [100–102].

The fact that these data indicate temporal alignment in interaction in both speech and gross trunk movement has interesting methodological implications, insofar as these data are derived from quite different sources and temporal windows. Both sets of data converge in indicating alignment. Speech alignment reflects fine control over syntactic, semantic and prosodic structures given form by a complex of articulatory mechanisms, whereas trunk movement reflects much more gross postural changes. The convergence shown by these results suggests that when coordination takes place, it operates simultaneously in different behavioural dimensions, being traceable in motor behaviour and speech as well as, presumably, other domains.

From a phonetic point of view, these speech results are also noteworthy in that they extend previous findings in English speakers to a Spanish-speaking sample, suggesting a possibly universal quality for periodicity in Q+A pairs, periodicity that can be manifested in different parameters of the speech signal. They also confirm the notion that, unlike other phenomena learned by cultural transmission, temporal entrainment stands as a basic biological phenomenon integral to all types of cooperative joint action [103, 104].

### Limitations and future directions

The present findings do not distinguish between the impact of positive affect and engagement from the musical activity itself. Although both MI and HB groups undertook activities—musical improvisation and cooperative use of building blocks—that were designed to promote affiliative attitudes, measurement of the extent to which these activities resulted in positive emotional states and interpersonal attitudes would have clarified the bases for the effects on interpersonal timing that were found. In addition, as causal relationships have been found between the affiliation dimension of social behaviour and musical harmonic coordination, musical temporal coordination, and the control (dominance) dimension [105], the analysis of musical interactions themselves is likely to be highly informative.

Choosing a task to compare musical improvisation is not straightforward. In our case, we used the tower task after significant piloting, since we found it to be an activity: (a) about as enjoyable and engaging as playing music; and (b) ecologically valid in social interactions. We consequently discarded alternative activities that promoted non-spontaneous interactions, although experimentally more controllable (e.g., synchronised pendulum movements or simultaneous throwing of a ball). However, building with blocks and musical improvisation differ in many other ways other than timing (e.g., pitch, differences in mental flow state, visual design aspects, and possibly others). Future research might consider exploring tasks with more accurate control of secondary variables.

Future research might reduce differences in potential anticipatory anxiety across groups by informing participants which condition they would be in only after the initial conversational phase. This design change alone could produce conflicting results in different participants: high anxiety after T1 in some, and release from tension during T2 in others. A better design option would be to randomly assign dyads to groups after T1, and also to inform them before they volunteered that they might be asked to play simple musical instruments. Consequences of these design choices could be worth exploring in further work, but they are incidental to the question of this experiment, namely: to what extent does improvising music together promote aligned conversational behaviour, compared with performing a cooperative but non-periodic task? Future work aimed at elucidating effects on non-musicians of being required to play music together, whether improvisational or not, could explore these and similar questions. Amongst other things, it could potentially contribute to a greater understanding of individual differences in reactions to group music making. This is turn could feed into the literature on joint music-making and mental health.

The relative effectiveness of our three measures of periodicity is of practical as well as theoretical interest. The 2interval criterion is the most stringent, and the AverageInterval measure probably the least stringent of our three. All three measures require a degree of periodicity across the turn space, but only the 2interval measure also requires periodicity within the Q. The 2interval measure is thus the only one of the three that includes the CA hypothesis that well-formed Qs become rhythmic towards their ends. Yet, in our study, the AverageInterval measure yielded the highest number of periodic Q+A pairs, and arguably the most statistically reliable results. Many questions are quite short (2 or 3 interpike intervals), so in practice there may be little difference between our AverageInterval measure and the more traditional sequential one. However, further research, of a different sort from that of the present study, is needed to confirm whether interlocutors entrain with the duration of the last interval in the Q, or its average interval, when the last interval is not similar to the average—that is, when the Q itself is not periodic in the traditional CA sense. Finally, only multi-interval Q+A pairs showed differences between our conditions, yet the total proportions of periodic Answer entries to single-interval and multi-interval Q+A pairs differed by only 5% (37% and 42%, respectively). These two findings suggest that although both kinds of Q+A pair may well provide sufficient temporal cues for interlocutors to enter the turn space in a coordinated manner, only multi-interval Q+A pairs are sensitive to variables such as those manipulated in the present study. In similar vein, while Early and On-Time A entry Q+A pairs returned no statistically significant differences, Late A entry Q+A pairs did for Group (MI and HB) and Conversation (T1 and T2), as well as their interaction, suggesting that Late entry Answers are more sensitive to the factors manipulated here.

A number of future experiments have already been mentioned, especially ones that manipulate the stage at which participants learn that they will be making music together. Differences between dyads of friends vs dyads of unknown participants could also be manipulated. It would be expected that interpersonal coordination (motor and speech) would be significantly higher between dyads of friends—in T2 but also, perhaps, already in T1—when compared to dyads of participants unknown to each other.

## Conclusions

The present study returned two main findings. First, overall, the data confirm our main hypothesis: musical interaction between non-musician participants unknown to each other does promote greater mutual behavioural alignment of body movement in subsequent conversational interaction than does a non-pulsed cooperative task. The second major finding corresponds to a partial, yet remarkable convergence between the motoric and speech dimensions of the effect of musical interaction on subsequent conversational interaction, despite the diverse anatomical areas and time windows such dimensions involve.

These findings lend weight to the idea that music and speech are culturally reconfigurable manifestations of the same universal human communicative ‘toolkit’ [16]. Interactive activity in one domain, music, appears to lead to enhanced interactive alignment in the other, speech. The effects of the musical interaction, despite its brevity, suggests that any “transfer effect” is neither a matter of practice in music-making, nor of formal training, nor of personal familiarity: the effect is found with musically untrained participants who are not known to each other prior to the musical interaction. And the effects are not bound to perception alone but are evident in production. Participants engaged in spontaneous conversation, involving not just perception (e.g., following body posture, being sensitive to periodicity in the partner’s speech) but also production (e.g., producing mirror-like movements in a fraction of a second, entering the turn-space on beat, etc.). All these results challenge strict cross-domain transfer effect paradigms and provide nuance to approaches that seek to elucidate the nature of human interaction by comparing speech and music [106].

## Supporting information

S1 Data (DOCX)

S2 Data (DOCX)

S3 Data (XLSX)

S1 File HB condition. (ZIP)

S2 File MI condition. (ZIP)

S3 File HB -T1. (ZIP)

S4 File HB -T2. (ZIP)
