# Correction: Musical improvisation enhances interpersonal coordination in subsequent conversation: Motor and speech evidence

**DOI:** 10.1371/journal.pone.0259704

**Published:** 2021-11-02

**Authors:** Juan Pablo Robledo, Sarah Hawkins, Carlos Cornejo, Ian Cross, Daniel Party, Esteban Hurtado

Fig 1 is incorrect. Please see the correct Fig 1 here.

**Fig 1:**
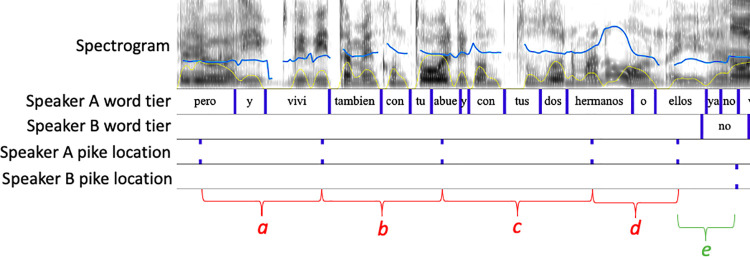
Screenshot of a Q+A pair set up in Praat. Top panel: spectrogram of both talkers’ speech (0–500 Hz), with speakers’ f0 contour (blue) and intensity contour (yellow) superimposed. Next two panels: speaker A and speaker B’s words, on separate tiers. (The compressed time scale means that the word *abuela* has been truncated to *abue*.) Lowest two panels: speaker A’s and speaker B’s pikes. The red letters *a*, *b*, *c* and *d* at the bottom of the figure denote the four (pike-to-pike) time intervals between the five pikes in the Q. The green letter e denotes the interval between the last pike of the Q and the first one in the A (across the turn space). The total duration of the speech displayed is 2.9 s.

The fourth sentence of the second paragraph under the heading Participants in the Materials and methods section, and the third and last sentences of the third paragraph under the heading Procedure in the Materials and methods section should have cited reference 49 instead of 54. The first sentence of the second paragraph of the Conclusions section should have cited reference 13 instead of 16.

The correct sentences should read:

“The musical interactions typically took place as a series of fairly short bouts (as in [49]) interspersed with preparatory or reflective episodes, rather than extending continuously through the time allotted.”

“As Hawkins, Cross & Ogden [49: 321–3] had found that the specific instructions given to participants substantially affected the extent to which participants (same-sex friends) engaged cooperatively and successfully in their musical task, we carried out a series of pilot studies in order to develop appropriate instructions for our participants, who were all same-sex strangers.”

“As in [49], we aimed to be as explicit as was required for the interaction to be cooperative and naturalistic while avoiding over-prescriptiveness by providing them with an easily understood common goal.”

“These findings lend weight to the idea that music and speech are culturally reconfigurable manifestations of the same universal human communicative ‘toolkit’ [13].”

The degrees of freedom are missing from the correlations reported in the fifth sentence of the first paragraph under the heading Distribution of Q+A pairs in the Results section. The correct sentence should read: “However, Pearson correlations between the number of Q+A pairs and the duration of the conversation were only significant for MI in T2 (r(13) = .80, p = 0.001). Correlations were non-significant for HB in both T1 and T2, and for MI in T1: r(11) = .45, p = 0.124; r(11) = .56, p = 0.073; and r(13) = .41, p = 0.164 respectively.”

There is an error in the last sentence of the sixth paragraph under the heading Periodicity of Q+A pairs in the Results section. The correct sentence should read: “However, in contrast with all other measures, the predicted increase for MI results is marginally significant: MI, T2 > T1, paired t(12) = -2.16, p = 0.052.”

There is an error in reference 54. The correct reference is: Ogden R, Hawkins S. Entrainment as a basis for co-ordinated actions in speech. In: The Scottish Consortium for ICPhS 2015, (Ed.). Proceedings of the 18th International Congress of Phonetic Sciences. Glasgow, UK: The University of Glasgow; ISBN 978-0-85261-941-4. Paper number 0599.

There is an error in reference 83. The correct reference is: Borrie SA, Liss JM. Rhythm as a coordinating device: Entrainment with disordered speech. J Speech, Language, and Hearing Research. 2014;57(3):815–24.

The following error was introduced during typesetting:

There is an error in reference 11. The correct reference is: Cross I. Music and communication in music psychology. Psychology of Music. 2014;42(6):809–19.

## References

[1] Robledo JP, Hawkins S, Cornejo C, Cross I, Party D, Hurtado E (2021) Musical improvisation enhances interpersonal coordination in subsequent conversation: Motor and speech evidence. PLoS ONE 16(4): e0250166. 10.1371/journal.pone.0250166 33857238 PMC8049323

