# Supplementary material for: Musical improvisation enhances interpersonal coordination in subsequent conversation: Motor and speech evidence
Source: PLoS One. 2021 Apr 15;16(4):e0250166. doi: 10.1371/journal.pone.0250166 (PMC8049323; doi:10.1371/journal.pone.0250166)
Supplement: S1 Data — (DOCX) [file pone.0250166.s001.docx]

**Musical improvisation enhances interpersonal coordination in subsequent conversation: Motor and speech evidence**

**Motion capture data files used in this study**

**1. Data files**

The *NaturalPoint Prime-41* motion capture system used in this study was supplied with the proprietary *Motive* software suite for calibrating the optical system, recording motion, and performing 3D reconstruction. In order to provide access to our data without requiring the purchase of a Motive software license, we supply our recordings in text files with comma separated values (CSV) format. These CSV files are the exact same ones we used as a starting for our mocap analyses, and were exported directly from Motive. This document describes their format.

# **2. Format**

As described in the article text, motion capture recordings at two different times were performed during each single experimental session. This yielded two recordings per session, corresponding to two CSV data files. Each filename ends with a “T1” or “T2” suffix, indicating whether the file corresponds to the first (time 1) or second (time 2) recording in a session. Text before those suffixes identifies the session, and the corresponding couple of participants.

The files we supply are exactly as exported from Motive software, except that rows have been already selected in order to trim recordings to the exact analysed time periods. The resulting format extends typical comma separated values by adding an initial text line with metadata. This is clear text that shows the Motive software version, recording and export framerates and other metadata. Only the export framerate was relevant for analyses. A second line is empty. Third line is the CSV header. Data types and headers are present until the 7^th^ line, and can be ignored. Data starts at 8^th^ row. First column is a frame number. Second column is time in seconds. Following columns are X, Y, Z triplets, each corresponding to the position of a marker detected in the frame. Units are meters, which is not relevant since Pearson cross-correlations pre-scale data by standard deviation. It must not be assumed that numbers in the same three columns in different frames correspond to the same marker; spatial proximity is the right criterion to track a marker from one frame to the next.
