# Supplementary material for: Musical improvisation enhances interpersonal coordination in subsequent conversation: Motor and speech evidence
Source: PLoS One. 2021 Apr 15;16(4):e0250166. doi: 10.1371/journal.pone.0250166 (PMC8049323; doi:10.1371/journal.pone.0250166)
Supplement: S2 Data — (DOCX) [file pone.0250166.s002.docx]

**Musical improvisation enhances interpersonal coordination in subsequent conversation: Motor and speech evidence**

**Speech data used in this study**

**1. Data**

Speech analyses were based on the temporal location of pikes. Accordingly, the CSV file contains the temporal location of all pikes included in the 179 Q+A pairs final corpus.

# **2. Format**

Each row in the CSV file corresponds to a given pike, and columns to information concerning the latter. The first three columns are related to the experimental design. The first column from left to right (named ‘Condition’) indicates whether the pikes belongs to the speech of participants in the Musical Improvisation (MI) or Hands-Busy (HB) groups. The second column (‘Dyad’) indicates the particular dyad by means of its unique number (e.g. 203, 226, etc.). As described in the article text, the experimental design meant that dyads had a first conversation (T1) and a second one (T2); the third column (‘Conversation’) indicates to which conversation a particular pike corresponds to.

The next two columns are related to the Q+A pair a given pike was located in. Within each dyad, a unique code name was attributed to each of its Q+A pars (e.g. ‘completo’), as indicated in the ‘Keyword’ column. Each Q+A pair was also given a unique identifying number within the corpus— 179 in total —, as indicated in the ‘Q+A abs#’ column.

The last three columns provide information exclusively concerning the pike corresponding to a given row. The ‘Q/A’ column indicates whether the pike was located in the question or the answer of a Q+A pair, and the ‘Pike#’ column specifies the ordinal location of the pike amid the rest of the pikes in the Q (e.g. the third pike in the question). The final row indicates the pike’s temporal location in second, with 0 corresponding to the start of the sound file.
